# Very Low Energy Ketogenic Therapy Effects on Fibrosis-Dependent Metabolic Reprogramming: A Serum NMR Pilot Study

**DOI:** 10.3390/nu18121950

**Published:** 2026-06-17

**Authors:** Rossella Donghia, Biagia Musio, Maria De Luca, Francesco Balestra, Giorgia Panzetta, Stefano Todisco, Pietro Mastrorilli, Sergio Coletta, Martina Di Chito, Gianluigi Giannelli, Vito Gallo, Maria Principia Scavo

**Affiliations:** 1 Data Science Unit, National Institute of Gastroenterology IRCCS “S. de Bellis”, Research Hospital, Via Turi 27, Castellana Grotte, 70013 Bari, Italy; rossella.donghia@irccsdebellis.it; 2 Dipartimento di Ingegneria Civile, Ambientale, del Territorio, Edile e di Chimica, Politecnico di Bari, Via Orabona 4, 70125 Bari, Italy; stefano.todisco@poliba.it (S.T.); pietro.mastrorilli@poliba.it (P.M.); vito.gallo@poliba.it (V.G.); 3 Laboratory of Molecular Medicine, National Institute of Gastroenterology IRCCS “S. de Bellis”, Research Hospital, Via Turi 27, Castellana Grotte, 70013 Bari, Italy; francesco.balestra@irccsdebellis.it (F.B.); giorgia.panzetta@irccsdebellis.it (G.P.); maria.scavo@irccsdebellis.it (M.P.S.); 4 Innovative Solutions S.r.l.—Spin-Off Company of the Polytechnic University of Bari, Zona H 150/B, Noci, 70015 Bari, Italy; 5 Core Facility Biobank, National Institute of Gastroenterology IRCCS “S. de Bellis”, Research Hospital, Via Turi 27, Castellana Grotte, 70013 Bari, Italy; sergio.coletta@irccsdebellis.it; 6 Center of Nutrition for the Research and the Care of Obesity and Metabolic Diseases, National Institute of Gastroenterology IRCCS “S. de Bellis”, Research Hospital, Via Turi 27, Castellana Grotte, 70013 Bari, Italy; martina.dichito@irccsdebellis.it; 7 Scientific Direction, National Institute of Gastroenterology IRCCS “S. de Bellis”, Research Hospital, Via Turi 27, Castellana Grotte, 70013 Bari, Italy; gianluigi.giannelli@irccsdebellis.it

**Keywords:** VLEKT, NMR spectroscopy, serum metabolomics, liver fibrosis, metabolic reprogramming

## Abstract

Background/Objectives: Ketogenic diets induce profound metabolic reprogramming; however, their impact on systemic metabolism in patients with hepatic fibrosis remains insufficiently characterized. This study aimed to investigate serum metabolic changes induced by Very Low Energy Ketogenic Therapy (VLEKT) and to assess the influence of hepatic fibrosis development on these metabolic adaptations. Methods: Fifty serum samples from 25 obese patients were analyzed using 1D 1H CPMG NMR spectroscopy at baseline (T0) and after 8 weeks of VLEKT (T1). Patients were stratified according to the FIB-E fibrosis index into a low-risk group (LR; FIB-E < 8) and an intermediate-high-risk group (IHR; FIB-E ≥ 8) for hepatic fibrosis onset. Results: An integrated approach combining NMR-based metabolomics and pathway enrichment analysis revealed a marked metabolic shift following VLEKT, characterized by increased ketone bodies (including β-hydroxybutyric acid and acetone), together with changes in amino acids and lipid-related signals. Among these, acetone provided a robust and quantifiable NMR signal, consistent with enhanced ketogenesis. Stratified analysis indicated differential metabolic flexibility: LR patients exhibited enhanced modulation of tricarboxylic acid (TCA) cycle-related metabolites, whereas IHR patients showed persistent alterations in aromatic amino acids and lipid signals. Significant correlations between tyrosine and β-alanine with clinical biochemical markers further supported the presence of a fibrosis-dependent metabolic signature. Conclusions: These findings highlight the potential of circulating metabolites as sensitive and non-invasive indicators of hepatic vulnerability and determinants of metabolic adaptability to VLEKT. Moreover, the study underscores the value of NMR-based metabolomics as an innovative tool for improving the non-invasive assessment of metabolic and hepatic health.

## 1. Introduction

Obesity represents one of the most pressing global public health challenges and is widely recognized as a true epidemic [1]. It is closely associated with systemic metabolic dysfunctions, particularly insulin resistance, type 2 diabetes mellitus (T2D), and metabolic dysfunction-associated steatohepatitis (MASLD), contributing to a substantial clinical and socioeconomic burden [2,3]. Within this spectrum, hepatic alterations may remain subclinical for long periods yet significantly influence systemic metabolic homeostasis and disease progression [4].

Dietary interventions inducing rapid weight loss and metabolic profile improvement have gained increasing clinical interest. Among these, Very Low Energy Ketogenic Therapy (VLEKT), a structured evolution of the traditional ketogenic diet, and Very Low-Calorie Ketogenic Diet (VLCKD) have emerged as effective nutritional strategies for obesity management. These approaches combine severe carbohydrate restriction with controlled caloric intake to induce nutritional ketosis, promoting a metabolic shift from glucose dependence to a predominant use of stored fats [5,6,7,8].

Biochemically, this metabolic transition is accompanied by an increase in the hepatic production of ketone bodies derived from the oxidation of fatty acids, a reduction in insulin levels, and the activation of lipolysis [9].

The liver plays a central role in coordinating these adaptations, reducing carbohydrate availability and limiting glycolysis and pyruvate production, with a remodeling of the Krebs cycle and mitochondrial redox balance. Meanwhile, increased β-oxidation boosts acetyl-CoA production, driving ketogenesis and supporting energy production through mitochondrial oxidative pathways [10,11]. In the case of efficient metabolic adaptation to ketosis, this adaptation is associated with a “protein sparing” effect, characterized by reduced energy use of amino acids and preservation of fat-free mass [12]. Nevertheless, this adaptation may vary depending on individual metabolic flexibility and hepatic functional status. In this context, aromatic amino acids and those involved in ketogenic and glucogenic pathways may serve as sensitive indicators of systemic and hepatic metabolic health [13].

Although VLEKT is effective in improving anthropometric and metabolic parameters, its systemic metabolic effects are not uniform across individuals [14,15]. In particular, the presence of subclinical hepatic alterations, such as fibrosis, may modulate metabolic flexibility and influence the ability to adapt to ketosis. However, the extent to which hepatic fibrosis shapes the systemic metabolic response to ketogenic interventions remains poorly understood. Conventional clinical markers and non-invasive techniques, including transient elastography, such as FibroScan, allow for rapid and reproducible assessment of liver stiffness and provide an indirect estimate of steatosis. These methods are currently essential tools in clinical practice for screening and monitoring metabolic liver diseases [16,17]. In addition to instrumental assessment, non-invasive composite indices have been developed to facilitate the stratification of liver fibrosis risk even in the absence of obvious clinical signs of liver disease. Among these, the FIB-E is a useful tool for identifying patients with different levels of fibrosis risk. In particular, as outlined in the guidelines of the European Association for the Study of the Liver (EASL), patients with a FIB-E index < 8 are classified as low risk (LR) to develop chronic liver disease, while those with a FIB-E index ≥ 8 are classified as intermediate-high risk (IHR) [18,19]. Nevertheless, such approaches are limited in capturing the complexity of metabolic adaptations at the systemic level.

In this framework, metabolomics offers a powerful approach to investigate integrated metabolic responses [20,21]. Nuclear magnetic resonance (NMR)-based metabolomics enables a comprehensive and quantitative characterization of circulating metabolites, allowing the simultaneous assessment of lipid, amino acid, and carbohydrate metabolism across multiple biochemical pathways. This analytical platform is highly reproducible, non-destructive, and inherently quantitative, supporting the robust profiling of a wide range of metabolites in complex biological fluids [21,22]. Importantly, metabolomic profiling allows for the detection of subtle metabolic perturbations and provides insights into dynamic metabolic changes associated with physiological and pathological conditions [23]. In the context of liver disease and nutrition, metabolomics has emerged as a valuable tool for identifying non-invasive biomarkers reflecting hepatic function. In addition to capturing metabolic responses to dietary interventions, it supports patient stratification and the development of personalized nutritional strategies [24,25].

In the present pilot study, metabolomic analysis was performed using nuclear magnetic resonance (NMR) spectroscopy, which enables a comprehensive and quantitative evaluation of the metabolic profile, providing integrated insights into lipid-, carbohydrate-, and amino acid-based metabolic pathways [26]. This powerful methodology facilitates the detection of subtle systemic perturbations and the identification of novel metabolic signatures linked to nutritional responses and hepatic status, thereby refining patient stratification and enabling personalized dietary interventions [25].

The systemic metabolic reprogramming induced by an 8-week VLEKT intervention was investigated in a cohort of 25 obese individuals. Longitudinal NMR-based metabolomic profiling with fibrosis risk stratification was used to assess how baseline hepatic status modulates the metabolic response to VLEKT. By integrating metabolomic and clinical data, this pilot study aimed to identify fibrosis-dependent metabolic signatures and serum NMR-derived features associated with hepatic vulnerability and metabolic flexibility.

## 2. Materials and Methods

### 2.1. Patients

A total of 30 patients were enrolled in this prospective pilot study, conducted over eight weeks at the Nutrition Centre for Obesity and Metabolic Diseases, National Institute of Gastroenterology “Saverio de Bellis” (Castellana Grotte, Bari, Italy). Of these, 25 eligible participants, adults aged 18–64 years with a BMI ≥ 30 kg/m^2^ and not receiving pharmacological or nutraceutical treatments, completed the study and were included in the final analysis. The study cohort comprised both sexes, including 14 females and 11 males, corresponding to 56% and 44%, respectively. All underwent a rigorously standardized 8-week VLEKT protocol, in accordance with established guidelines [27].

Stringent exclusion criteria encompassed excessive alcohol intake (>20 g/day women; >30 g/day men), verified via direct anamnesis per American and European guidelines. Further exclusions included type 1 (T1D) and type 2 (T2D) diabetes mellitus, severe cardiovascular/respiratory diseases, gastrointestinal/renal disorders, psychiatric conditions, active infections, substance abuse, eating disorders, and VLEKT contraindications. Rare metabolic disorders such as porphyria, carnitine deficiencies, carnitine palmitoyl transferase/translocase deficiencies, pyruvate carboxylase deficiency, and mitochondrial fatty acid oxidation defects were also excluded. Smoking, pregnancy, and breastfeeding were documented during clinical interviews.

The protocol received local Medical Ethics Committee approval (No. 179/C.E. de Bellis, 13 May 2022), adhering to the Declaration of Helsinki. Written informed consent was obtained from all participants pre-enrolment, with registration on ClinicalTrials.gov (NCT05477212, First Posted: 28 July 2022). Recruitment spanned July 2022–December 2023, featuring clinical/biochemical evaluations at baseline (T0) and post-intervention (T1) following VLEKT.

### 2.2. Dietary Intervention

All participants adhered to a structured 8 weeks of VLEKT providing 650–800 kcal/day, characterized by a low carbohydrate intake (<30 g/day, derived mainly from low glycemic index vegetables) and a low-fat intake (approximately 20 g/day of lipids, derived predominantly from extra virgin olive oil, during the initial phase). Permitted foods encompassed low-glycaemic index, low-sugar vegetables with recommended fiber intake, aromatic herbs and spices, extra virgin olive oil (limited to ≤2 tablespoons/day), and lemon as a condiment.

During the first phase of 8 weeks, participants were asked to consume at least two liters of water per day. Given the restrictive and potentially unbalanced nature of the diet, micronutrient supplementation was provided throughout the intervention period. In the second phase of 2 weeks of diet, a small increase in calorie intake was permissible while maintaining the ketogenic macronutrient proportions. Dietary adherence was rigorously monitored through food diaries that clinicians specifically requested from all participating patients.

### 2.3. Anthropometric and Clinical Assessment

Anthropometric measurements were collected at the start of the study (T0) and after eight weeks of treatment with VLEKT (T1). Body weight and height were determined using the same calibrated scale and stadiometer for all participants, and body mass index (BMI) was calculated as weight in kilograms divided by height in meters squared (kg/m^2^).

Body composition was assessed by bioelectrical impedance analysis (BIA) using a single-frequency bioimpedance analyser (BIA-101, 50 kHz; Akern Bioresearch, Florence, Italy), according to standardized procedures [28]. Fat mass (FM, expressed in kilograms and percentage) and fat-free mass (FFM, expressed in kilograms and percentage) were derived from the impedance measurements using validated predictive equations provided by the manufacturer. To minimize variability related to hydration status, participants were asked to fast for at least 12 h and to avoid physical activity for 24 h before the examination.

Venous blood samples were collected between 8:00 and 9:00 a.m. after an overnight fast. Fasting plasma glucose (mg/dL), fasting serum insulin (µIU/mL), glycated hemoglobin (HbA1c, %), triglycerides (mg/dL), total cholesterol (mg/dL), high-density lipoprotein cholesterol (HDL-C, mg/dL), and low-density lipoprotein cholesterol (LDL-C, mg/dL) were measured using standardized automated laboratory methods. Hepatic biochemical parameters included aspartate aminotransferase (AST, U/L), alanine aminotransferase (ALT, U/L), and gamma-glutamyl transferase (GGT, U/L).

Insulin resistance was estimated using the homeostasis model assessment for insulin resistance (HOMA-IR), calculated as fasting insulin (µIU/mL) multiplied by fasting glucose (mg/dL) divided by 405. Renal function was assessed by measuring serum uric acid (mg/dL), creatinine (mg/dL), and estimating the glomerular filtration rate (GFR, mL/min). All biochemical analyses were performed in the same certified clinical laboratory to ensure analytical consistency. An aliquot of serum obtained at each time point was immediately stored at −80 °C until subsequent metabolomic analyses.

Liver status about hepatic steatosis and fibrosis was assessed using transient elastography (FibroScan^®^, Echosens, Paris, France), a non-invasive ultrasound-based technique capable of quantifying liver stiffness and steatosis. The tests were performed after a minimum 6 h fasting period. Liver stiffness was expressed in kilopascals (kPa) using M or XL probes as appropriate, while steatosis was assessed using the controlled attenuation parameter (CAP, expressed in dB/m). Subjects were placed in the supine position with their right arm raised, and measurements were obtained from the right hepatic lobe via the intercostal approach. Patients with a FIB-E score < 8 were classified as LR, while those with a score ≥ 8 were categorized as IHR for developing chronic liver disease.

### 2.4. Preparation of Serum Samples for NMR Analysis

A total of 50 serum samples (25 paired samples) were analyzed. Samples were collected from 25 patients at two time points: baseline (T0), before the initiation of the VLEKT, and after 8 weeks of dietary intervention (T1) and stored at −80 °C until analysis. For NMR measurements, 150 µL of frozen serum was thawed at room temperature for 1 h. Subsequently, 350 µL of a 0.010 M sodium azide (NaN_3_) solution prepared in deuterium oxide (D_2_O) was added. The mixture was vortexed at 2400 rpm for 1 min using an Advanced Vortex Mixer ZX3 (VELP Scientifica Srl, Usmate Velate, Italy). The resulting clear solution was transferred into a 5 mm NMR tube for spectroscopic analysis. NaN_3_ (CAS No. 26628-22-8; ≥99.5%, Sigma-Aldrich, Milan, Italy) and D_2_O (CAS No. 7789-20-0; ≥99.9% D, Sigma-Aldrich, St. Louis, MO, USA) were used for sample preparation. NMR tubes (Norell 509-UP-7) were supplied by Norell (Landisville, NJ, USA).

### 2.5. NMR Experiments and Data Processing

NMR spectra were acquired on a Bruker Avance I 400 MHz spectrometer (Bruker BioSpin GmbH, Rheinstetten, Germany) equipped with a 5 mm inverse broadband (BBI) probe. To minimize baseline distortion caused by macromolecular signals and to improve spectral comparability across samples, a one-dimensional ^1^H NMR experiment combining a Carr–Purcell–Meiboom–Gill (CPMG) T2 filter and water signal presaturation was employed. Spectra were acquired using the pulse sequence (cpmgpr1D) with the following parameters: FID size (64 k), spectral width (14 ppm), transmitter offset (4.702 ppm), 90° pulse (10.12 µs), pre-saturation band width (25 Hz), dummy scans (16), number of scans (128), acquisition time (5.86 s), T_2_ filter loops (128), relaxation delay (3 s). Spectra were acquired using TopSpin 2.1 software (Bruker BioSpin GmbH, Rheinstetten, Germany), including automatic sample loading, 5 min temperature equilibration, locking to D_2_O, tuning, matching, and shimming.

Raw Free Induction Decays (FIDs) were processed using MestReNova 11.0 (Mestrelab Research SL, Santiago de Compostela, Spain). Processing steps included: zero-filling to 128 k data points; Fourier transformation with exponential line broadening (0.1 Hz); manual phase correction; automatic baseline correction; chemical shift calibration using the glucose doublet at δ = 5.24 ppm.

Spectra were pre-treated before performing the chemometric analysis upon segmentation into 0.002 ppm-wide buckets in the range 9.00–0.70 ppm. Water region (5.17–4.69 ppm) was excluded. Bucket integrals were normalized to total spectral intensity and, subsequently, UV-scaled (mean-centered and divided by the square root of the standard deviation of each variable).

Quantification of tyrosine and β-alanine was performed from 1D ^1^H CPMG NMR spectra through a targeted approach based on metabolite-specific signal integration. Preliminary external calibration curves were constructed for both metabolites using standard solutions at increasing concentrations, establishing a linear relationship between signal intensity and metabolite concentration. Signal quality was evaluated using the signal-to-noise ratio (S/N), and limits of detection (LOD) were determined for tyrosine and β-alanine during the calibration procedure. These parameters were used to ensure the reliability and robustness of the targeted quantification approach adopted in this study.

An internal standard such as trimethylsilylpropionic acid (TSP) was intentionally not used. Although widely employed in NMR metabolomics, TSP is known to interact with serum proteins, which may cause signal broadening and chemical shift variations, ultimately compromising quantitative accuracy in protein-rich biofluids. To avoid these matrix-related artifacts, quantification was performed using an external calibration approach, following established methodological considerations for NMR-based metabolomics of biological samples [29].

For tyrosine, characteristic aromatic proton signals in the δ 6.8–7.2 ppm region were selected, while β-alanine was quantified based on methylene proton signals in the δ ~2.5–3.2 ppm region. Signal intensities were determined by integrating the area under the curve (AUC) within these predefined spectral regions. Quantitative values were obtained by applying the calibration curves to the integrated peak areas, allowing conversion of signal integral into concentration estimates.

The resulting values were used as relative metabolite concentration estimates for subsequent statistical and correlation analyses.

### 2.6. Statistical Analysis

Continuous variables are expressed as median and interquartile range (IQR). Differences between paired observations (baseline, T0, and post-intervention, T1) were assessed using the Wilcoxon signed-rank test. Comparisons between independent groups (FIB-E < 8 vs. FIB-E ≥ 8) were performed using the Wilcoxon rank-sum test.

For metabolomic data, both univariate and multivariate analyses were performed using MetaboAnalyst 6.0. Univariate analysis was conducted by combining Fold Change (FC ≥ 2) with Student’s *t*-test (*p* < 0.05) as implemented in MetaboAnalyst to identify significantly altered metabolites between T0 and T1, as well as to assess differences between fibrosis-stratified groups (FIB-E < 8 vs. ≥8) at each time point.

Multivariate analysis included Principal Component Analysis (PCA) and Partial Least Squares Discriminant Analysis (PLS-DA). Model performance was evaluated using explained variance (R^2^) and predictive ability (Q^2^) and assessed by permutation testing (*n* = 1000) to estimate model validity and avoid overfitting.

Spearman correlation analysis, based on Spearman’s rank correlation coefficient (ρ), was used to evaluate associations between metabolite levels (tyrosine and β-alanine) and anthropometric, metabolic, and hepatic parameters. This non-parametric approach was selected due to the relatively small sample size and the non-normal distribution of metabolomic and clinical variables, ensuring a robust assessment of monotonic relationships without assuming linearity.

All statistical tests were two-sided, and a *p*-value < 0.05 was considered statistically significant. Given the exploratory nature of this pilot study, no correction for multiple testing was applied, and results should be interpreted as hypothesis-generating. This approach prioritizes sensitivity and exploratory discovery over strict control of false positives.

The analyses were conducted with StataCorp. 2025. Stata Statistical Software: Release 19. College Station, TX: StataCorp LLC., and RStudio (“Globemaster Allium” Release).

## 3. Results

### 3.1. Effects of VLEKT on Anthropometric, Metabolic, and Hepatic Parameters in the Total Cohort

In the total cohort of 25 obese patients treated with VLEKT for 8 weeks, marked improvements were observed across key anthropometric, metabolic, and hepatic parameters, as detailed in Table 1. Notably, BMI significantly decreased from 44.10 to 39.20 kg/m^2^ (*p* < 0.0001), accompanied by a marked reduction in FM from 55.50 to 45.70 kg (*p* < 0.0001), while FFM remained unchanged (*p* = 0.54). Regarding glycemic control, insulin levels significantly decreased from 26.90 to 14.90 μIU/mL (*p* = 0.01), along with a reduction in HOMA index from 6.00 to 2.77 (*p* = 0.01). HbA1c also showed a significant decrease (5.30 vs. 5.60, *p* = 0.0003), whereas fasting blood glucose did not change significantly (*p* = 0.13).

Lipid profile improved overall, with significant reductions in triglycerides (79.90 vs. 113.00; *p* = 0.04), LDL cholesterol (98.10 vs. 126.60, *p* = 0.0009), and total cholesterol (160.00 vs. 190.00, *p* = 0.0009). However, HDL cholesterol significantly decreased from 45.00 to 38.50 (*p* = 0.0009). Liver-related parameters showed notable improvements, with a significant reduction in GGT (17.00 vs. 31.00, *p* < 0.0001), CAP (260.00 vs. 323.00, *p* = 0.0002), and FIB-E from 8.60 to 4.90 (*p* < 0.0001).

Renal function remained stable, with no significant differences in creatinine (*p* = 0.40) or GFR (*p* = 0.63), although uric acid levels slightly increased (*p* = 0.01).

As shown in Table S1, both female and male participants exhibited clinically relevant improvements in parameters recorded. In females, significant reductions were observed in BMI (*p* = 0.0001) and FM (*p* = 0.003), accompanied by a decrease in HbA1c (*p* = 0.003), and improvements in lipid profile, including reductions in LDL (*p* = 0.01) and total cholesterol (*p* = 0.01), as well as an increase in HDL (*p* = 0.002). Liver-related markers also improved, with significant reductions in GGT (*p* = 0.002), CAP score (*p* = 0.01), and fibrosis index (FIB-E, *p* = 0.0001). No significant changes were observed in fasting glucose, HOMA, or uric acid levels. In males, similar but less consistently statistically significant results were observed. Significant reductions were found in BMI (*p* = 0.001), FM (*p* = 0.001), GGT (*p* = 0.01), CAP score (*p* = 0.01), and FIB-E (*p* = 0.001). Improvements in glycemic and lipid parameters were evident but did not reach statistical significance, including HbA1c, triglycerides, LDL, and total cholesterol. Insulin and HOMA also showed favorable reductions, although without statistical significance. No meaningful changes were observed in other parameters.

### 3.2. NMR-Based Metabolomic Changes Following VLEKT in the Total Cohort

In the total clinical cohort, an integrated metabolomic analysis combining univariate and multivariate approaches was performed to characterize metabolic alterations in patients before and after VLEKT. As a first step, the overall spectral profile was visually inspected through stacked 1D 1H CPMG NMR spectra (Figure S1), followed by metabolite assignment of the observed resonances (Table S2). A representative ^1^H CPMG NMR spectrum is provided in the Supplementary Material (Figure S2), with detailed annotation of key metabolites. A comprehensive NMR-based metabolomic analysis enabled the identification of a wide range of metabolites spanning organic acids, amino acids, carbohydrates, and lipid-related compounds, each characterized by specific chemical shifts and multiplicity patterns. Univariate analysis, based on the combined application of Fold Change (FC ≥ 2) and Student’s *t*-test (*p* < 0.05), identified several metabolites significantly altered between T0 and T1, as listed in Table 2. According to the univariate results reported in Table 2, a consistent pattern of reciprocal variation was observed. Most metabolites exhibited lower levels at T0 and increased levels at T1, while a smaller subset showed the opposite trend. Notably, ketone bodies such as acetone were significantly increased after the intervention, reflecting enhanced ketogenesis. β-hydroxybutyrate (BHB) signals were also identified in the spectra (see Table S2 for chemical shift assignments). However, due to partial signal overlap and the limited chemical shift dispersion at 400 MHz, accurate and reliable quantification of BHB was not feasible under the present experimental conditions. Consequently, acetone, which exhibited well-resolved and quantifiable signals, was used as a surrogate marker of ketogenesis.

*N*-acetyl glycoproteins, were increased at T1, whereas metabolites associated with glycolysis and energy metabolism, including acetoacetate and fumaric acid, were reduced, together with a decrease in LDL/VLDL-related signals. Additionally, significant modulation of amino acid levels was observed, with decreases in phenylalanine and increases in branched-chain and glucogenic amino acids (e.g., valine, lysine, glutamine).

**Figure 1:**
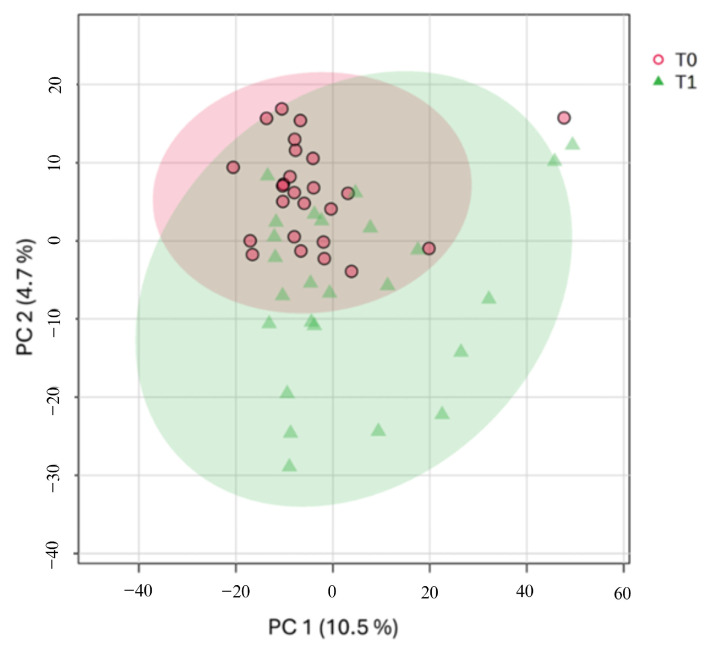
PCA scores plot of 1D 1H CPMG NMR data showing samples at T0 (red circles) and T1 (green triangles). Explained variance for each principal component is reported in brackets. Ellipses represent the 95% confidence intervals for each group.

Principal Component Analysis (PCA) revealed only a partial separation between T0 and T1 samples, with substantial overlap along the principal components, indicating that timepoint-related differences account for only a limited fraction of the total variance (PC1 = 10.5%, PC2 = 4.7%). As shown in the scores plot (Figure 1), T0 samples appear relatively clustered in a narrower region, whereas T1 samples exhibit a markedly broader dispersion. The confidence ellipses of the two groups largely overlap, confirming the absence of a clear separation between timepoints.

This distribution pattern suggests that, while some degree of variation is present, such differences are not sufficient to define a strong global discriminative metabolic signature, with most of the variability being shared across samples. PLS-DA as a supervised multivariate model was explored to evaluate potential class discrimination between T0 and T1 samples. Although this approach suggested a certain degree of separation between groups (Figure S3a), model validation indicated limited reliability. In particular, the permutation test (*n* = 1000) demonstrated that the model performance was not significantly different from random classification (*p* = 0.996), providing strong evidence of overfitting and lack of model validity (Figure S3b).

These findings indicate that no clear global metabolic separation was observed between the two conditions, with most variability shared across samples. To further investigate whether metabolic differences were present within specific subgroups, subjects were stratified according to fibrosis status (FIB-E).

This stratification allowed a more detailed longitudinal and intergroup assessment of metabolic changes and enabled the identification of subgroup-specific adaptations that were not evident in the global PCA model. Since supervised multivariate models, including PLS-DA, did not show adequate statistical validity, subsequent interpretation was primarily based on univariate analysis. This approach was adopted to reduce the risk of overfitting and to support a more reliable identification of biologically relevant metabolic alterations.

### 3.3. NMR-Based Metabolomic Stratified Analysis by Fibrosis Status (FIB-E)

To investigate fibrosis-dependent metabolic differences and their modulation following VLEKT, both baseline (T0) and post-intervention (T1) intergroup comparisons were performed using univariate analysis.

At baseline, patients with FIB-E ≥ 8 exhibited a distinct metabolic profile compared with those with FIB-E < 8 (Table 3), characterized by significantly higher levels of ethanol, tyrosine, and β-alanine (all *p* < 0.01), along with altered phenylalanine levels. These metabolites showed marked fold changes, with β-alanine (FC = 0.42), ethanol (FC = 0.44), and tyrosine (FC = 0.47) indicating enrichment in the higher fibrosis group, consistent with perturbations in amino acid metabolism and hepatic metabolic function.

Ethanol signals observed in serum spectra may reflect both exogenous contamination and endogenous production. Indeed, ethanol contamination during biofluid sampling and handling has been reported even under standardized conditions [30]. However, endogenous ethanol is a recognized physiological phenomenon mediated by gut microbiota and detectable in circulation even in the absence of alcohol intake [31]. Moreover, altered endogenous ethanol production has been associated with metabolic disorders, including obesity and liver disease [32]. Although minor contributions from exogenous sources cannot be absolutely excluded, the consistent detection of ethanol signals and their differential distribution between fibrosis-stratified groups suggest that, at least in part, they may reflect underlying metabolic differences rather than random contamination alone.

In contrast, patients with FIB-E < 8 showed significantly higher levels of unsaturated lipids (FC = 20.7, *p* = 0.001), suggesting a more preserved lipid metabolic profile.

Following the ketogenic diet intervention (T1), intergroup differences remained evident, although the metabolic profiles evolved (Table 4). Patients with FIB-E < 8 exhibited significantly higher levels of *N*-acetyl glycoproteins, acetone, 1-methylhistidine, and LDL/VLDL, reflecting enhanced ketogenesis, protein turnover, and lipid transport. In contrast, patients with FIB-E ≥ 8 showed significantly higher levels of phenylalanine (FC = 0.26, *p* = 0.015) and citric acid (FC = 0.46, *p* = 0.039), indicating persistent alterations in amino acid metabolism and incomplete metabolic reprogramming at the level of central energy pathways.

To better contextualize these metabolite-level differences, pathway enrichment analysis was performed separately on metabolites significantly altered and identified through univariate analysis (FC ≥ 2, Student’s *t*-test *p* < 0.05) at T0 and T1, to distinguish fibrosis-dependent metabolic alterations from diet-induced metabolic adaptations. To ensure biological interpretability of pathway results, metabolite selection was restricted to compounds that could be reliably mapped to defined metabolic networks. Accordingly, composite NMR signals (e.g., lipoproteins and glycoproteins) were excluded, and priority was given to key metabolites involved in interconnected pathways. While the T1 univariate analysis identified a subset of significantly altered metabolites (e.g., phenylalanine, citrate, lactate), additional biologically relevant metabolites detected at baseline—particularly tyrosine and β-alanine—were included in pathway reconstruction due to their central role within the same metabolic networks. This integrative approach reflects the network-based nature of pathway analysis and might allow a more comprehensive representation of fibrosis-associated metabolic perturbations beyond single timepoint statistical thresholds.

**Figure 2:**
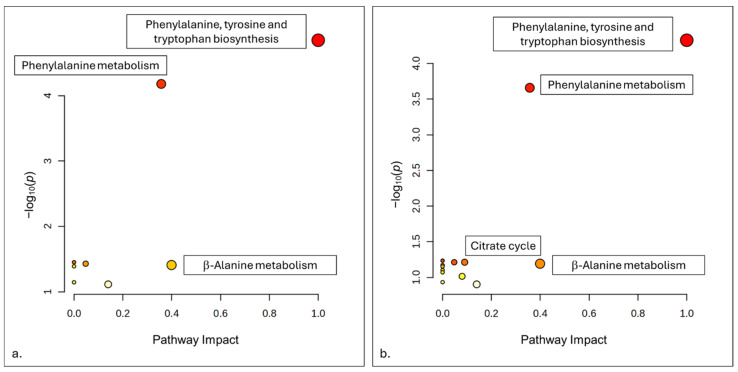
Comparative bubble plots of pathway enrichment analysis performed at baseline (T0, panel (**a**)) and after VLEKT intervention (T1, panel (**b**)) in fibrosis-stratified groups. Pathway enrichment was based on significantly altered metabolites identified through univariate analysis and biologically relevant metabolites selected for pathway reconstruction. The x-axis represents pathway impact (topological importance), while the y-axis shows statistical significance expressed as −log_10_(p). Bubble size is proportional to pathway impact; color intensity reflects increasing statistical significance.

At T0, enrichment analysis revealed a clear perturbation of aromatic amino acid metabolism, with phenylalanine, tyrosine and tryptophan biosynthesis emerging as the most significantly impacted pathway (*p* ≈ 10^−5^, pathway impact = 1), followed by phenylalanine metabolism and β-alanine metabolism. These results were driven by the consistent alteration of phenylalanine, tyrosine, and β-alanine, indicating that fibrosis-related metabolic dysregulation is already established at baseline and is primarily centered on amino acid metabolism (Figure 2a).

Following VLEKT (T1), pathway analysis confirmed the persistence of this metabolic signature, with phenylalanine, tyrosine and tryptophan biosynthesis and phenylalanine metabolism remaining the most significantly enriched pathways. In addition, pathways related to energy metabolism, including the TCA cycle and glycolysis/pyruvate metabolism, emerged, reflecting the metabolic shift induced by ketogenic intervention (Figure 2b).

### 3.4. Anthropometric and Metabolic Changes Stratified by Fibrosis Status (FIB-E)

Building on the metabolomic alterations observed in fibrosis-stratified groups, we next evaluated whether these molecular differences were reflected in distinct clinical and anthropometric trajectories. In particular, the subgroup-specific metabolic signatures identified through NMR analysis prompted further investigation into the extent to which fibrosis status modulates the systemic response to VLEKT at the clinical level.

After stratification by fibrosis status, the effects of VLEKT were first evaluated longitudinally within each subgroup and subsequently compared between groups at baseline (T0) and post-intervention (T1). In the total cohort, after the VLEKT intervention, a significant improvement was observed in several anthropometric, metabolic, and hepatic parameters (Table 1). When stratified by baseline FIB-E (≥8 vs. <8), both groups experienced significant reductions in BMI and FM (all *p* ≤ 0.003) (Table 5). In the FIB-E ≥ 8 group, greater metabolic improvements were observed. Specifically, significant reductions were found in HOMA index (*p* = 0.04), HbA1c (*p* = 0.003), LDL cholesterol (*p* = 0.002), and total cholesterol (*p* = 0.002), whereas these changes were not statistically significant in the FIB-E < 8 group. Liver-related outcomes improved in both groups, although with different patterns. GGT significantly decreased in both groups (*p* = 0.01 and *p* = 0.002, respectively). CAP showed a significant reduction only in the FIB-E < 8 group (*p* = 0.001), while the decrease in the FIB-E ≥ 8 group did not reach statistical significance (*p* = 0.06). FIB-E significantly improved in both groups, with a greater absolute reduction in the FIB-E ≥ 8 group (−4.35 vs. −1.80; *p* for between-group difference = 0.004). HDL cholesterol significantly decreased only in the FIB-E ≥ 8 group (*p* = 0.01), while triglycerides did not significantly change in either group. Renal parameters remained largely unchanged in both groups, although uric acid significantly increased in the FIB-E ≥ 8 group (*p* = 0.01). A borderline difference between groups was observed for GFR changes (*p* = 0.05).

### 3.5. NMR-Based Correlation Analysis of Tyrosine and β-Alanine with Anthropometric and Biochemical Parameters

Considering the fibrosis-dependent metabolic alterations identified in Section 3.3 and the subgroup-specific clinical responses to VLEKT described in Section 3.4, particular attention was directed toward metabolites that emerged as key discriminants across both molecular and clinical levels. Among these, tyrosine and β-alanine consistently showed significant modulation between fibrosis groups and across timepoints, highlighting their potential role as integrative markers of systemic and hepatic metabolic adaptation.

The reliability of the quantitative data was supported by analytical performance parameters (S/N and LOD), as summarized in Table S3 (Supplementary Material).

To further explore this hypothesis, correlation analyses were performed to investigate the association of tyrosine and β-alanine with anthropometric and biochemical parameters, aiming to clarify their relationship with metabolic control and liver-related outcomes.

**Figure 3:**
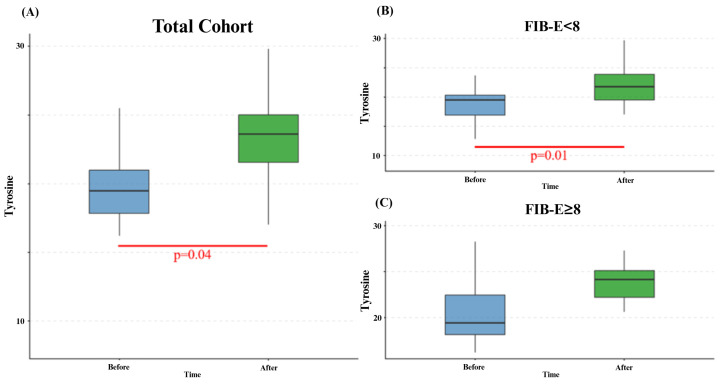
Changes in serum tyrosine levels (expressed as ppm) following 8-week VLEKT intervention. (**A**) Total cohort (*n* = 25): significant decrease post-VLEKT (*p* = 0.04). (**B**) FIB-E < 8 subgroup (*n* = 11): significant reduction post-VLEKT (*p* = 0.01). (**C**) FIB-E ≥ 8 subgroup (*n* = 14). Data are presented as boxplots showing median and interquartile range. Statistical significance was assessed using the Wilcoxon signed-rank test.

Tyrosine levels showed significant changes following the intervention. In the total cohort, a modest but significant decrease was observed after VLEKT (*p* = 0.04) (Figure 3A). This effect was more pronounced in the FIB-E < 8 subgroup, where tyrosine significantly decreased after treatment (*p* = 0.01) (Figure 3B), suggesting a more effective metabolic adaptation in patients with lower fibrosis rescue.

**Figure 4:**
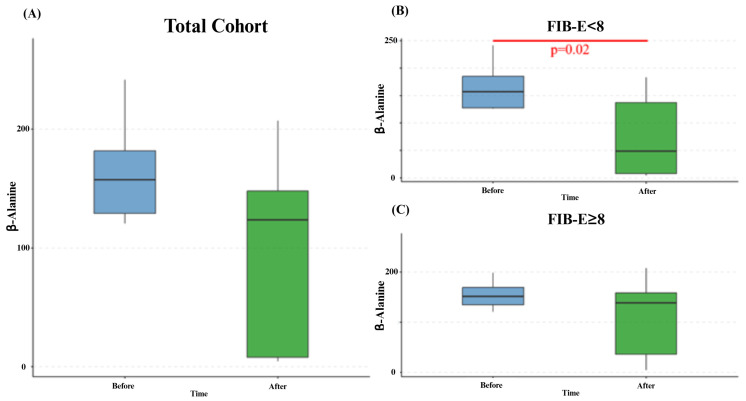
Changes in serum β-alanine levels (expressed as ppm) following 8-week VLEKT intervention. (**A**) Total cohort (*n* = 25). (**B**) FIB-E < 8 subgroup (*n* = 11): significant reduction post-VLEKT (*p* = 0.02). (**C**) FIB-E ≥8 subgroup (*n* = 14). Data are presented as boxplots showing median and interquartile range. Statistical significance was assessed using the Wilcoxon signed-rank test.

A similar pattern was observed for β-alanine, although its modulation appeared more selective. While no significant variation was detected in the overall cohort, β-alanine levels significantly decreased in the FIB-E < 8 group (*p* = 0.02) (Figure 4B), indicating a subgroup-specific response consistent with the metabolic flexibility observed in patients with lower fibrosis risk.

Spearman correlation analyses revealed distinct patterns according to fibrosis severity and timepoints. In the FIB-E < 8 group, Tyrosine (Table 6) showed a significant positive correlation with fasting blood glucose at baseline (ρ = 0.66, *p* = 0.03). At follow-up, significant positive correlations emerged with BMI (ρ = 0.63, *p* = 0.04) and FM (ρ = 0.67, *p* = 0.02). No significant associations were observed for changes (Δ/Δ correlations). In the FIB-E ≥ 8 group, tyrosine was positively correlated with blood glucose both at baseline (ρ = 0.60, *p* = 0.02) and longitudinally (T0/T1: ρ = 0.52, *p* = 0.05), as well as with HOMA index over time (ρ = 0.64, *p* = 0.03). At follow-up, significant positive associations were observed with BMI (ρ = 0.60, *p* = 0.02), GGT (ρ = 0.55, *p* = 0.04), and uric acid levels (ρ = 0.55, *p* = 0.04), while a negative correlation emerged with HDL cholesterol (ρ= −0.61, *p* = 0.02). Notably, changes in tyrosine (Δ/Δ) were inversely correlated with BMI (ρ = −0.54, *p* = 0.04) and glomerular filtration rate (GFR) (ρ = −0.61, *p* = 0.02), and positively correlated with creatinine (ρ = 0.70, *p* = 0.006) in the FIB-E ≥ 8 group.

In contrast, β-alanine showed generally weaker and less consistent association patterns (Table 7). In the FIB-E < 8 group, β-alanine showed a strong negative correlation with HDL cholesterol over time (T0/T1: ρ = −0.77, *p* = 0.01). Additionally, a positive correlation was observed between β-alanine and uric acid longitudinally (ρ = 0.76, *p* = 0.01). Borderline associations were noted with creatinine at baseline (ρ = 0.62, *p* = 0.05) and ALT (ρ = 0.60, *p* = 0.07). In the FIB-E ≥ 8 group, β-alanine was positively correlated with total cholesterol at baseline (ρ = 0.55, *p* = 0.04) and with creatinine both at baseline (ρ = 0.58, *p* = 0.03) and longitudinally (ρ = 0.60, *p* = 0.02). At follow-up, a strong positive correlation was observed with FIB-E (ρ = 0.70, *p* = 0.007), along with a positive correlation with uric acid (ρ = 0.60, *p* = 0.03). Conversely, changes in β-alanine were negatively associated with GGT (ρ= −0.58, *p* = 0.03) in the FIB-E ≥ 8 group.

## 4. Discussion

Consistent with accumulating evidence, the present study demonstrates that VLEKT induces a deep metabolic remodeling in obese patients, further supporting findings from prospective studies showing its efficacy in promoting rapid adipose tissue mobilization and enhancing metabolic flexibility [14]. This effect extends beyond simple body weight loss, encompassing improvements in insulin sensitivity, lipid profile, liver-related indices, along with a mitochondrial metabolic reorganization and enhanced hepatic energy [33]. NMR-based serum metabolomic profiling provided a comprehensive and integrative readout of this metabolic transition, revealing a coordinated shift from glucose-dependent metabolism toward increased lipid oxidation and ketone body production. This is supported by the marked increase in acetone at the cohort level, a key circulating ketone body reflecting enhanced hepatic ketogenesis, together with the reduction in lactate and phenylalanine levels, indicating effective engagement of metabolic reprogramming and a remodeling of central energy pathways [34]. Importantly, these results suggest that VLEKT acts as a systemic metabolic reprogramming strategy rather than merely a caloric restriction approach. Indeed, the concomitant modulation of amino acids and intermediates of mitochondrial metabolism, including lysine, valine, glutamine, and citric acid, points to a broader reorganization of metabolic networks. This coordinated response is consistent with enhanced mitochondrial efficiency and a shift toward oxidative metabolism, highlighting the capacity of ketogenic interventions to reshape systemic metabolic homeostasis.

A major finding of this study is that baseline status substantially influences both the magnitude and the quality of the metabolic response. LR patients exhibited a more efficient adaptation, characterized by modulation of TCA cycle-related metabolites such as citrate, suggesting improved mitochondrial flexibility. In contrast, IHR patients retained a more altered metabolic profile, particularly in amino acid and lipid signals. These findings suggest that subclinical hepatic vulnerability may limit the capacity to fully adapt to ketosis.

Within this context, tyrosine and β-alanine emerged as key fibrosis-sensitive metabolites. The higher baseline levels of tyrosine observed in patients with increased fibrosis risk are consistent with impaired hepatic clearance of aromatic amino acids, a well-known feature of reduced liver metabolic efficiency. This finding aligns with previous evidence linking elevated aromatic amino acids to hepatic dysfunction and progression of metabolic liver disease. Similarly, β-alanine may reflect perturbations in nitrogen handling and amino acid catabolism, providing an additional layer of information on the metabolic phenotype associated with liver vulnerability [35]. Importantly, the combined alteration of these metabolites suggests that the response to VLEKT is shaped not only by ketogenesis but also by the pre-existing hepatic metabolic state. Notably, the persistence of altered phenylalanine after intervention supports the presence of a pre-existing disturbance in amino acid metabolism linked to hepatic dysfunction that may not be fully normalized by the short-term ketogenic therapy. Correlation analyses reinforce these findings, revealing distinct association patterns according to fibrosis severity. In IHR patients, tyrosine showed consistent correlations with markers of metabolic impairment, including insulin resistance, glycemic parameters, and liver-related indicators. These associations suggest that tyrosine may serve as an integrative marker of both systemic metabolic dysregulation and hepatic vulnerability. Moreover, its relationship with BMI and FM at follow-up highlights a link between residual adiposity and altered amino acid metabolism, further supporting its role in reflecting metabolic quality beyond mere weight loss. In contrast, β-alanine exhibited weaker and more context-dependent association patterns. While significant correlations were observed with selected parameters such as HDL cholesterol, uric acid, and renal function markers, these associations were less consistent across timepoints and fibrosis groups. This suggests that β-alanine contributes to the metabolic phenotype; its role is more nuanced and may reflect specific aspects of metabolic adaptation rather than a global marker of dysfunction.

These results highlight that the metabolic response to VLEKT is not uniform but is strongly shaped by the underlying hepatic condition. In this context, serum-based molecular profiling approaches represent a powerful tool to capture systemic biological responses. Indeed, circulating biofluids provide an integrated snapshot of physiological and pathological processes, enabling the identification of clinically relevant molecular signatures. Evidence from recent studies supports the utility of serum-derived molecular profiling approaches, including both vesicle-associated and metabolite-based signatures, in capturing clinically relevant perturbations and supporting biomarker discovery in complex clinical settings [23,24,36]. Within this framework, serum NMR metabolomics provides a sensitive and integrative platform to capture these coordinated metabolic adaptations, offering insights that are not accessible through conventional clinical measurements alone [20,22]. While non-invasive tools such as elastography and fibrosis indices effectively assess structural liver alterations, metabolomic profiling adds a functional dimension by revealing how the organism responds to dietary intervention at the molecular level.

Several limitations should be considered when interpreting these findings. First, the study included a relatively small sample size, as expected for a pilot study. Therefore, these findings require validation in larger cohorts with longer observation and, ideally, external replication. Second, the follow-up period was short, so the durability of the metabolic and hepatic improvements remains uncertain, as does their long-term clinical significance. In addition, future studies should assess whether tyrosine and β-alanine may predict response to VLEKT or track liver vulnerability over time.

Nevertheless, the clinical implications of these findings are particularly relevant in the management of obesity. Inter-individual variability in metabolic adaptability represents a major determinant of therapeutic success, and the identification of fibrosis-dependent metabolic signatures may contribute to a more precise stratification of patients. In addition, individuals with subclinical liver vulnerability may require tailored nutritional strategies or longer intervention periods to achieve optimal metabolic reprogramming. The integration of metabolomic biomarkers such as tyrosine and β-alanine into clinical practice may improve monitoring of treatment response and early identification of metabolic resilience or fragility.

## 5. Conclusions

This study provides preliminary evidence that VLEKT induces a coordinated and clinically meaningful metabolic reprogramming in obese patients, extending beyond weight loss to encompass systemic metabolic and hepatic adaptations. Notably, the metabolic response appears to be strongly modulated by baseline fibrosis status, highlighting the role of hepatic condition in shaping metabolic flexibility during ketogenic interventions. The identification of tyrosine and β-alanine as fibrosis-sensitive metabolites underscores their potential as non-invasive indicators of hepatic metabolic vulnerability and adaptive capacity. Overall, serum NMR metabolomics emerges as a promising complementary tool to conventional clinical assessment, offering new opportunities for precision nutritional phenotyping and personalized therapeutic monitoring in obesity.

## Figures and Tables

**Table 1:** Anthropometric, metabolic, and hepatic parameters before and after VLEKT in the total cohort (*n* = 25). Data are presented as median (interquartile range, IQR). Statistical significance between timepoints was assessed using the Wilcoxon signed-rank test (*p* ^).

Parameters	Before	After	*p* ^^^
BMI (Kg/m^2^)	44.10 (13.40)	39.20 (12.70)	<0.0001
FM (Kg)	55.50 (32.30)	45.70 (28.45)	<0.0001
FFM (Kg)	63.75 (22.55)	61.35 (26.85)	0.54
Blood Sugar (mg/dL)	91.00 (8.00)	87.00 (11.00)	0.13
Insulin (UI/mL)	26.90 (24.70)	14.90 (7.15)	0.01
HOMA	6.00 (6.17)	2.77 (1.86)	0.01
HbA1c (mmol/mol)	5.60 (0.70)	5.30 (0.50)	0.0003
Triglycerides (mg/dL)	113.00 (107.00)	79.90 (46.00)	0.04
HDL (mg/dL)	45.00 (12.80)	38.50 (9.10)	0.0009
LDL (mg/dL)	126.60 (27.60)	98.10 (60.00)	0.0009
Total Cholesterol (mg/dL)	190.00 (34.00)	160.00 (55.00)	0.0009
AST (U/L)	24.00 (13.00)	22.00 (14.00)	0.99
ALT (U/L)	28.00 (38.00)	26.00 (24.00)	0.31
GGT (U/L)	31.00 (36.00)	17.00 (16.00)	<0.0001
CAP (dB/m)	323.00 (95.00)	260.00 (100.00)	0.0002
FIB-E	8.60 (6.80)	4.90 (3.90)	<0.0001
Uricemia (mg/dL)	6.10 (2.30)	6.40 (2.90)	0.01
Creatininemia (mg/dL)	0.83 (0.24)	0.83 (0.26)	0.40
GFR (mL/min)	91.00 (5.00)	87.00 (14.00)	0.63

Abbreviations: BMI, Body Mass Index; FM, Fat Mass; FFM, Fat Fee Mass; HOMA, Homeostatic Model Assessment; HbA1c, Glycated Hemoglobin; HDL, High-Density Lipoprotein; LDL, Low-Density Lipoprotein; AST, Aspartate Aminotransferase; ALT, Alanine Aminotransferase; GGT, Gamma-Glutamyl Transferase; CAP, Controlled Attenuation Parameter; FIB-E, Fibroscan Elastography; GFR, Glomerular Filtration Rate.

**Table 2:** List of significant spectral features identified through combined Fold Change (FC) analysis and univariate statistical testing (Student’s *t*-test) comparing T0 and T1 samples. Unadjusted *p*-value; values < 0.05 considered significant (no multiple testing correction applied). Chemical shift regions (δ, ppm) correspond to annotated metabolites based on 1D 1H CPMG NMR assignment. FC values, calculated as [T0]/[T1], describe the magnitude and direction of variation, where FC > 1 indicates higher relative signal intensity at T0 and FC < 1 indicates higher relative intensity at T1. Arrows indicate relative abundance at each time point (↑ higher, ↓ lower). Abbreviations: LDL, low-density lipoprotein; VLDL, very low-density lipoprotein.

d (ppm)	Metabolite	FC	Raw. *p*-Value	T0	T1
0.791845–0.793845	LDL/VLDL	20.122	0.000220	↑	↓
1.01985–1.02185	Valine	0.336	0.004000	↓	↑
1.23785–1.23985	LDL/VLDL	21.603	0.000680	↑	↓
1.77785–1.77985	Lysine	0.356	0.000190	↓	↑
2.03385–2.03585	*N*-acetyl glycoproteins	28.518	0.008300	↑	↓
2.16585–2.16785	Glutamine	0.472	0.019980	↓	↑
2.21585–2.21785	Acetone	0.461	0.024520	↓	↑
2.30385–2.30585	Acetoacetate	20.347	0.113000	↑	↓
2.48585–2.48785	Glutamine	0.471	0.010390	↓	↑
2.52385–2.52585	Citric acid	0.471	0.010770	↓	↑
3.29385–3.29585	Glycerophosphocholine	0.499	0.029710	↓	↑
5.44185–5.44385	Unsaturated lipid	0.487	0.030470	↓	↑
6.67785–6.67985	Fumaric acid	22.371	0.043090	↑	↓
7.55785–7.55985	Phenylalanine	21.975	0.001150	↑	↓

**Table 3:** List of significant spectral features identified through combined Fold Change (FC) analysis and univariate statistical testing (Student’s *t*-test) comparing patient groups stratified by fibrosis index (FIB-E < 8 vs. FIB-E ≥ 8) at T0. Unadjusted *p*-value; values < 0.05 considered significant (no multiple testing correction applied). Chemical shift regions (δ, ppm) correspond to metabolite assignments based on 1D 1H CPMG NMR spectral annotation. FC values, calculated as [FIB-E < 8]/[FIB-E ≥ 8], describe the magnitude and direction of variation, where FC > 1 indicates higher relative signal intensity in the FIB-E < 8 group and FC < 1 indicates higher relative intensity in the FIB-E ≥ 8 group. Arrows indicate relative abundance in each group (↑ higher, ↓ lower).

d (ppm)	Metabolite	FC	Raw. *p*-Value	FIB-E < 8	FIB-E ≥ 8
1.18144–1.18344	Ethanol	0.440	0.00077528	↓	↑
5.28144–5.28344	Unsaturated lipid	20.719	0.0012244	↑	↓
6.91344–6.91544	Tyrosine	0.469	0.0063918	↓	↑
3.19544–3.19744	b-alanine	0.424	0.0064517	↓	↑
7.53944–7.54144	Phenylalanine	36.544	0.0194	↑	↓

**Table 4:** List of significant spectral features identified through combined Fold Change (FC) analysis and univariate statistical testing (Student’s *t*-test) comparing patients stratified by fibrosis index (FIB-E < 8 vs. FIB-E ≥ 8) at T1. Unadjusted *p*-value; values < 0.05 considered significant (no multiple testing correction applied). Chemical shift regions (δ, ppm) correspond to metabolite assignments based on 1D 1H NMR spectral annotation. FC and log2(FC) values, calculated as [FIB-E < 8]/[FIB-E ≥ 8], describe the magnitude and direction of variation, where FC > 1 indicates higher relative signal intensity in the FIB-E < 8 group and FC < 1 indicates higher relative intensity in the FIB-E ≥ 8 group. Arrows indicate relative abundance in each group (↑ higher, ↓ lower). Abbreviations: LDL, low-density lipoprotein; VLDL, very low-density lipoprotein.

d (ppm)	Metabolite	FC	Raw. *p*-Value	FIB-E < 8	FIB-E ≥ 8
2.01144–2.01344	*N*-acetyl glycoproteins (Nac)	21.654	0.0033451	↑	↓
2.21944–2.22144	Acetone	24.227	0.03424	↑	↓
7.03544–7.03744	1-Methylhistidine	21.611	0.011951	↑	↓
7.53544–7.53744	Phenylalanine	0.262	0.01539	↓	↑
7.72344–7.72544	1-Methylhistidine	20.904	0.018449	↑	↓
0.927444–0.929444	LDL/VLDL	2.079	0.035026	↑	↓
2.52544–2.52744	Citric acid	0.456	0.039361	↓	↑

**Table 5:** Anthropometric, metabolic, and hepatic parameters before and after VLEKT, stratified by baseline fibrosis status (FIB-E < 8 vs. FIB-E ≥ 8). Data are presented as median and interquartile range. Within-group changes were assessed using the Wilcoxon signed-rank test (*p* ^). Between-group comparisons were performed using the Wilcoxon rank-sum test for baseline values (*p* ¥) and for changes over time (Δ, *p* †).

	FIB-E (<8)				FIB-E (≥8)					
Parameters	(*n* = 11)				(*n* = 14)					
	Before	After	ΔAfter − Before	*p* ^	Before	After	ΔAfter − Before	*p* ^	*p* ¥	*p* †
BMI (Kg/m^2^)	34.20 (6.90)	32.00 (5.70)	−3.40 (1.20)	0.001	48.55 (6.90)	45.30 (7.40)	−4.75 (2.50)	0.0001	<0.0001	0.08
FM (Kg)	34.90 (17.90)	27.90 (23.10)	−9.20 (8.00)	0.001	68.20 (15.50)	56.90 (10.00)	−12.60 (10.00)	0.003	<0.0001	0.22
FFM (Kg)	57.10 (20.40)	54.40 (13.40)	−2.90 (5.40)	0.99	72.40 (33.60)	80.10 (32.80)	−1.50 (5.80)	0.58	0.002	0.85
Blood Sugar (mg/dL)	91.00 (10.00)	90.00 (18.00)	−4.00 (9.00)	0.34	93.00 (14.00)	84.60 (7.00)	−2.00 (19.80)	0.39	0.38	0.56
Insulin (UI/mL)	15.60 (23.36)	11.10 (9.51)	−6.49 (21.70)	0.23	27.60 (22.10)	15.80 (12.60)	−14.82 (20.10)	0.06	0.09	0.34
HOMA	3.29 (5.34)	2.14 (2.29)	−1.91 (5.79)	0.23	8.04 (8.03)	3.27 (3.06)	−5.39 (7.05)	0.04	0.04	0.07
HbA1c (mmol/mol)	5.30 (0.70)	5.40 (0.60)	−0.10 (0.10)	0.06	5.65 (0.50)	5.25 (0.50)	−0.50 (0.30)	0.003	0.29	0.01
Triglycerides (mg/dL)	89.00 (67.00)	87.00 (50.00)	−17.00 (80.00)	0.55	128.50 (110.00)	78.95 (48.00)	−37.00 (54.00)	0.06	0.49	0.21
HDL (mg/dL)	47.30 (17.80)	40.50 (7.30)	−8.70 (10.40)	0.06	38.15 (16.80)	36.10 (11.00)	−3.85 (5.20)	0.01	0.08	0.09
LDL (mg/dL)	133.60 (21.60)	114.40 (40.00)	−21.60 (37.60)	0.23	121.80 (30.00)	81.80 (23.60)	−36.00 (29.40)	0.002	0.12	0.13
Total Cholesterol (mg/dL)	203.00 (21.00)	183.00 (42.00)	−27.00 (56.00)	0.23	183.50 (41.00)	141.50 (47.00)	−38.50 (21.00)	0.002	0.35	0.24
AST (U/L)	18.00 (8.00)	18.00 (6.00)	1.00 (7.00)	0.99	25.00 (17.00)	25.50 (17.00)	−0.50 (11.00)	0.99	0.18	0.56
ALT (U/L)	21.00 (26.00)	18.00 (20.00)	−4.00 (11.00)	0.23	39.50 (39.00)	27.50 (35.00)	−1.00 (18.00)	0.99	0.12	0.36
GGT (U/L)	20.00 (15.00)	16.00 (12.00)	−7.00 (10.00)	0.01	34.50 (40.00)	24.00 (20.00)	−10.50 (10.00)	0.002	0.39	0.78
CAP (dB/m)	267.00 (86.00)	225.00 (97.00)	−81.00 (56.00)	0.001	349.00 (94.00)	314.00 (99.00)	−41.50 (52.00)	0.06	0.04	0.12
Uricemia (mg/dL)	5.50 (2.60)	6.00 (1.20)	0.60 (2.00)	0.55	6.45 (2.00)	7.80 (3.40)	0.80 (1.60)	0.01	0.18	0.68
Creatininemia (mg/dL)	0.83 (0.28)	0.83 (0.31)	0.06 (0.07)	0.06	0.82 (0.23)	0.82 (0.28)	−0.005 (0.14)	0.77	0.88	0.25
GFR (mL/min)	89.00 (11.00)	77.00 (16.00)	−4.00 (13.00)	0.18	91.00 (2.00)	91.00 (9.00)	0.00 (6.00)	0.73	0.10	0.05

Abbreviation: BMI, Body Mass Index; FM, Fat Mass; FFM, Fat Fee Mass; HOMA, Homeostatic Model Assessment; HbA1c, Glycated Hemoglobin; HDL, High-Density Lipoprotein; LDL, Low-Density Lipoprotein; AST, Aspartate Aminotransferase; ALT, Alanine Aminotransferase; GGT, Gamma-Glutamyl Transferase; CAP, Controlled Attenuation Parameter; FIB-E, Fibroscan Elastography; GFR, Glomerular Filtration Rate.

**Table 6:** Spearman correlation analysis between tyrosine levels and anthropometric, metabolic, and hepatic parameters stratified by fibrosis status (FIB-E < 8 vs. ≥8). Values are expressed as Spearman’s rank correlation coefficient (ρ) and *p*-value [ρ (p)] at baseline (T0/T0), longitudinally (T0/T1), at follow-up (T1/T1), and for changes over time (Δ/Δ). Significant correlations (*p* < 0.05) are highlighted in bold.

Parameters	FIB-E (<8)				FIB-E (≥8)			
	T0/T0	T0/T1	T1/T1	Δ/Δ	T0/T0	T0/T1	T1/T1	Δ/Δ
BMI (Kg/m^2^)	0.24 (0.46)	0.24 (0.48)	**0.63 (0.04)**	0.55 (0.08)	−0.02 (0.95)	0.23 (0.41)	**0.60 (0.02)**	**−0.54 (0.04)**
FM (Kg)	0.18 (0.59)	0.28 (0.39)	**0.67 (0.02)**	0.17 (0.61)	0.04 (0.88)	0.16 (0.58)	0.46 (0.11)	−0.30 (0.31)
FFM (Kg)	0.14 (0.66)	0.01 (0.98)	−0.09 (0.79)	−0.005 (0.99)	−0.04 (0.88)	−0.14 (0.63)	**0.56 (0.05)**	−0.22 (0.46)
Blood Sugar (mg/dL)	**0.66 (0.03)**	0.43 (0.18)	−0.01 (0.98)	0.21 (0.53)	**0.60 (0.02)**	**0.52 (0.05)**	−0.09 (0.76)	0.01 (0.98)
Insulin (UI/mL)	−0.01 (0.98)	0.38 (0.24)	0.25 (0.44)	0.30 (0.36)	0.28 (0.33)	0.50 (0.07)	0.33 (0.24)	−0.22 (0.44)
HOMA	0.14 (0.68)	0.36 (0.27)	0.19 (0.56)	0.36 (0.27)	0.51 (0.09)	**0.64 (0.03)**	0.31 (0.32)	−0.07 (0.83)
HbA1c (mmol/mol)	−0.05 (0.88)	0.07 (0.83)	−0.16 (0.63)	0.15 (0.65)	0.40 (0.15)	0.25 (0.38)	−0.06 (0.84)	0.15 (0.60)
Triglycerides (mg/dL)	−0.34 (0.31)	0.14 (0.67)	0.17 (0.60)	0.10 (0.77)	0.27 (0.34)	0.36 (0.20)	0.19 (0.51)	−0.18 (0.54)
HDL (mg/dL)	0.00 (0.99)	−0.53 (0.09)	−0.32 (0.33)	0.25 (0.46)	−0.04 (0.90)	−0.08 (0.78)	**−0.61 (0.02)**	−0.27 (0.34)
LDL (mg/dL)	−0.14 (0.66)	0.25 (0.44)	−0.04 (0.89)	0.05 (0.87)	0.42 (0.13)	0.48 (0.08)	0.12 (0.67)	−0.31 (0.27)
Total Cholesterol (mg/dL)	−0.35 (0.28)	0.43 (0.19)	−0.04 (0.91)	−0.23 (0.49)	0.37 (0.19)	0.23 (0.42)	−0.03 (0.91)	−0.18 (0.54)
AST (U/L)	−0.07 (0.84)	0.19 (0.58)	0.24 (0.48)	−0.45 (0.16)	0.02 (0.93)	−0.16 (0.59)	0.21 (0.47)	0.28 (0.32)
ALT (U/L)	0.20 (0.54)	0.30 (0.36)	−0.01 (0.98)	−0.33 (0.32)	−0.06 (0.84)	0.03 (0.91)	0.34 (0.24)	0.13 (0.66)
GGT (U/L)	0.04 (0.89)	−0.04 (0.89)	−0.10 (0.76)	0.05 (0.88)	0.19 (0.50)	0.09 (0.75)	**0.55 (0.04)**	−0.18 (0.52)
CAP (dB/m)	0.24 (0.48)	0.33 (0.31)	0.05 (0.87)	0.02 (0.96)	−0.29 (0.32)	0.00 (0.99)	0.33 (0.24)	−0.22 (0.45)
Uricemia (mg/dL)	0.21 (0.53)	0.56 (0.07)	0.55 (0.08)	−0.17 (0.60)	0.14 (0.63)	−0.03 (0.93)	**0.55 (0.04)**	**0.54 (0.05)**
Creatininemia (mg/dL)	0.03 (0.93)	−0.01 (0.99)	−0.12 (0.72)	0.07 (0.83)	−0.01 (0.96)	−0.34 (0.22)	0326 (0.37)	**0.70 (0.006)**
GFR (mL/min)	0.26 (0.44)	0.19 (0.57)	0.18 (0.59)	−0.11 (0.74)	−0.10 (0.73)	0.47 (0.09)	−0.02 (0.93)	**−0.61 (0.02)**

Abbreviations: ρ, rho coefficient; BMI, Body Mass Index; FM, Fat Mass; FFM, Fat Fee Mass; HOMA, Homeostatic Model Assessment; HbA1c, Glycated Hemoglobin; HDL, High-Density. Lipoprotein; LDL, Low-Density Lipoprotein; AST, Aspartate Aminotransferase; ALT, Alanine Aminotransferase; GGT, Gamma-Glutamyl Transferase; CAP, Controlled Attenuation Parameter; GFR, Glomerular Filtration Rate.

**Table 7:** Spearman correlation analysis between β-alanine levels and anthropometric, metabolic, and hepatic parameters stratified by fibrosis status (FIB-E < 8 vs. ≥8). Values are expressed as Spearman’s rank correlation coefficient (ρ) and *p*-value [ρ (p)] at baseline (T0/T0), longitudinally (T0/T1), at follow-up (T1/T1), and for changes over time (Δ/Δ). Significant correlations (*p* < 0.05) are highlighted in bold.

Parameters	FIB-E (<8)				FIB-E (≥8)			
	T0/T0	T0/T1	T1/T1	Δ/Δ	T0/T0	T0/T1	T1/T1	Δ/Δ
BMI (Kg/m^2^)	−0.02 (0.96)	−0.07 (0.85)	0.19 (0.60)	0.29 (0.42)	−0.07 (0.81)	−0.27 (0.35)	0.14 (0.62)	0.03 (0.93)
FM (Kg)	−0.25 (0.48)	−0.17 (0.62)	0.13 (0.72)	0.49 (0.15)	−0.07 (0.80)	−0.26 (0.39)	0.24 (0.43)	0.26 (0.38)
FFM (Kg)	0.55 (0.10)	0.50 (0.14)	−0.05 (0.88)	−0.35 (0.31)	0.32 (0.28)	0.33 (0.27)	0.03 (0.91)	−0.34 (0.25)
Blood Sugar (mg/dL)	0.57 (0.09)	0.42 (0.22)	0.13 (0.72)	−0.25 (0.48)	0.09 (0.76)	0.31 (0.28)	−0.09 (0.74)	−0.10 (0.73)
Insulin (UI/mL)	0.01 (0.99)	0.33 (0.34)	0.37 (0.29)	0.32 (0.36)	−0.15 (0.60)	−0.47 (0.09)	0.34 (0.22)	−0.18 (0.53)
HOMA	0.10 (0.77)	0.41 (0.24)	0.30 (0.40)	0.28 (0.42)	−0.24 (0.45)	−0.53 (0.07)	0.28 (0.38)	−0.28 (0.37)
HbA1c (mmol/mol)	0.08 (0.81)	0.22 (0.54)	−0.07 (0.85)	0.07 (0.83)	0.31 (0.27)	0.39 (0.16)	−0.48 (0.08)	−0.24 (0.40)
Triglycerides (mg/dL)	0.01 (0.99)	0.27 (0.45)	0.13 (0.71)	0.57 (0.08)	0.49 (0.07)	−0.01 (0.96)	0.11 (0.70)	0.30 (0.30)
HDL (mg/dL)	−0.27 (0.44)	**−0.77 (0.01)**	−0.59 (0.07)	−0.24 (0.49)	−0.37 (0.18)	−0.04 (0.90)	−0.41 (0.15)	−0.40 (0.16)
LDL (mg/dL)	0.36 (0.30)	0.41 (0.24)	0.57 (0.08)	0.45 (0.18)	0.21 (0.47)	−0.07 (0.80)	−0.10 (0.73)	0.06 (0.84)
Total Cholesterol (mg/dL)	−0.07 (0.84)	0.57 (0.08)	0.59 (0.07)	0.27 (0.44)	**0.55 (0.04)**	−0.01 (0.99)	−0.03 (0.91)	0.36 (0.21)
AST (U/L)	0.22 (0.53)	0.20 (0.57)	−0.15 (0.67)	0.17 (0.63)	0.34 (0.22)	0.40 (0.15)	0.29 (0.31)	0.42 (0.13)
ALT (U/L)	0.60 (0.07)	0.45 (0.18)	0.10 (0.77)	0.50 (0.14)	0.36 (0.20)	0.27 (0.34)	0.42 (0.14)	0.21 (0.47)
GGT (U/L)	0.26 (0.46)	0.13 (0.71)	0.12 (0.73)	0.41 (0.24)	−0.12 (0.68)	0.02 (0.94)	0.51 (0.06)	**−0.58 (0.03)**
CAP (dB/m)	0.34 (0.32)	0.38 (0.28)	0.07 (0.84)	−0.30 (0.40)	0.18 (0.54)	−0.09 (0.74)	0.26 (0.37)	0.51 (0.06)
Uricemia (mg/dL)	0.59 (0.07)	**0.76 (0.01)**	0.32 (0.37)	0.47 (0.16)	−0.03 (0.91)	−0.05 (0.87)	**0.60 (0.03)**	0.34 (0.23)
Creatininemia (mg/dL)	**0.62 (0.05)**	0.42 (0.22)	−0.17 (0.63)	0.36 (0.30)	**0.58 (0.03)**	**0.60 (0.02)**	−0.04 (0.88)	0.01 (0.98)
GFR (mL/min)	−0.13 (0.71)	0.01 (0.99)	0.54 (0.11)	−0.11 (0.74)	−0.45 (0.11)	−0.38 (0.18)	0.15 (0.61)	0.10 (0.74)

Abbreviations: ρ, rho coefficient; BMI, Body Mass Index; FM, Fat Mass; FFM, Fat Fee Mass; HOMA, Homeostatic Model Assessment; HbA1c, Glycated Hemoglobin; HDL, High-Density Lipoprotein; LDL, Low-Density Lipoprotein; AST, Aspartate Aminotransferase; ALT, Alanine Aminotransferase; GGT, Gamma-Glutamyl Transferase; CAP, Controlled Attenuation Parameter; GFR, Glomerular Filtration Ratio.

## Data Availability

All data generated in this study are present in this manuscript.

## Supplementary Materials

The following supporting information can be downloaded at: https://www.mdpi.com/article/10.3390/nu18121950/s1.
