# Supplementary material for: Very Low Energy Ketogenic Therapy Effects on Fibrosis-Dependent Metabolic Reprogramming: A Serum NMR Pilot Study
Source: Nutrients. 2026 Jun 17;18(12):1950. doi: 10.3390/nu18121950 (PMC13304591; doi:10.3390/nu18121950)
Supplement: Supplementary file 1 [file nutrients-18-01950-s001.zip › nutrients-4357790-supplementary.pdf]

Supplementary Information

Very Low Energy Ketogenic Therapy Effect on Fibrosis-Dependent Metabolic Reprogramming: A Serum NMR Pilot Study

Rossella Donghia[1*], Biagia Musio[2*§], Maria De Luca[3§], Francesco Balestra[3], Giorgia Panzetta[3], Stefano Todisco[2], Pietro Mastrorilli[2,4], Sergio Coletta[5], Martina Di Chito[6], Gianluigi Giannelli[7], Vito Gallo[2,4ɸ] and Maria Principia Scavo[3ɸ]

1. Data Science Unit, National Institute of Gastroenterology IRCCS "S. de Bellis", Research Hospital, Via Turi 27, Castellana Grotte, 70013 Bari, Italy. rossella.donghia@irccsdebellis.it (R.D.)
2. Dipartimento di Ingegneria Civile, Ambientale, del Territorio, Edile e di Chimica, Politecnico di Bari, Via Orabona 4, 70125 Bari, Italy. biagia.musio@poliba.it (B.M.); stefano.todisco@poliba.it (S.T.); pietro.mastrorilli@poliba.it (P.M.); vito.gallo@poliba.it (V.G.)
3. Laboratory of Molecular Medicine, National Institute of Gastroenterology IRCCS "S. de Bellis", Research Hospital, Via Turi 27, Castellana Grotte, 70013 Bari, Italy. maria.deluca@irccsdebellis.it (M.D.L.); francesco.balestra@irccsdebellis.it (F.B.); giorgia.panzetta@irccsdebellis.it (G.P.); maria.scavo@irccsdebellis.it (M.P.S)
4. Innovative Solutions S.r.l.—Spin-Off Company of the Polytechnic University of Bari, Zona H 150/B, I-70015 Noci (BA), Italy
5. Core Facility Biobank, National Institute of Gastroenterology IRCCS "S. de Bellis", Research Hospital, Via Turi 27, Castellana Grotte, 70013 Bari, Italy; sergio.coletta@irccsdebellis.it (S.C);
6. Center of Nutrition for the Research and the Care of Obesity and Metabolic Diseases, National Institute of Gastroenterology IRCCS "S. de Bellis", Research Hospital, Via Turi 27, Castellana Grotte, 70013 Bari, Italy; martina.dichito@irccsdebellis.it (M.D.C.)
7. Scientific Direction, National Institute of Gastroenterology IRCCS "S. de Bellis", Research Hospital, Via Turi 27, Castellana Grotte, 70013 Bari, Italy. gianluigi.giannelli@irccsdebellis.it (G.G.)

* These authors have contributed equally to this work

ɸ These authors have contributed equally to this work

§ Corresponding Author: Maria De Luca (M.D.L.) maria.deluca@irccsdebellis.it; Biagia Musio (B.M.) biagia.musio@poliba.it

Table S1. Anthropometric, metabolic, and hepatic parameters before and after VLEKT in the cohort stratified by sex. Data are presented as median (interquartile range, IQR). Statistical significance between timepoints was assessed using the Wilcoxon signed-rank test (p^).

Parameters	Female (n=14)			Male (n=11)		
	Before	After	p ^	Before	After	p ^
BMI (Kg/m²)	42.95 (13.40)	40.15 (13.40)	0.0001	44.10 (18.10)	39.20 (14.20)	0.001
FM (Kg)	57.90 (23.90)	49.70 (24.90)	0.003	53.00 (36.70)	39.90 (27.80)	0.001
FFM (Kg)	55.80 (7.30)	53.30 (5.40)	0.99	72.40 (22.60)	80.10 (24.80)	0.55
Blood Sugar (mg/dL)	90.50 (10.00)	83.10 (8.00)	0.77	92.00 (22.00)	88.00 (16.00)	0.11
Insulin (UI/mL)	22.90 (22.47)	14.35 (7.46)	0.18	32.40 (29.20)	14.90 (12.95)	0.06
HOMA	4.80 (5.58)	3.00 (1.97)	0.15	7.62 (8.83)	2.61 (3.83)	0.06
HbA1c (mmol/mol)	5.45 (0.70)	5.05 (0.60)	0.003	5.70 (0.90)	5.50 (0.70)	0.06
Triglycerides (mg/dL)	75.00 (51.00)	74.00 (38.00)	0.42	178.00 (70.00)	115.00 (58.00)	0.06
HDL (mg/dL)	49.60 (19.80)	42.55 (12.60)	0.002	38.50 (11.80)	37.10 (4.60)	0.23
LDL (mg/dL)	125.70 (24.70)	90.10 (38.30)	0.01	144.20 (27.50)	110.10 (72.50.)	0.06
Total Cholesterol (mg/dL)	186.00 (22.00)	149.15 (49.00)	0.01	207.00 (36.00)	185.00 (87.00)	0.06
AST (U/L)	18.00 (9.00)	19.00 (7.00)	0.42	30.00 (9.00)	26.00 (17.00)	0.55
ALT (U/L)	19.50 (22.00)	20.85 (14.00)	0.99	56.00 (33.00)	33.00 (34.00)	0.23
GGT (U/L)	24.50 (22.00)	15.50 (13.00)	0.002	31.00 (39.00)	23.00 (29.00)	0.01
CAP (dB/m)	284.00 (98.00)	258.00 (124.00)	0.01	340.00 (100.00)	293.00 (138.00)	0.01
FIB-E	8.55 (7.00)	4.45 (4.30)	0.0001	9.40 (7.40)	5.10 (3.00)	0.001
Uricemia (mg/dL)	4.80 (2.80)	5.50 (3.20)	0.06	6.70 (1.30)	7.10 (1.90)	0.23
Creatininemia (mg/dL)	0.68 (0.18)	0.70 (0.15)	0.99	0.92 (0.17)	0.97 (0.15)	0.23
GFR (mL/min)	91.00 (2.00)	91.00 (2.00)	0.99	89.00 (10.00)	89.00 (10.00)	0.99

Abbreviations: BMI, Body Mass Index; FM, Fat Mass; FFM, Fat Fee Mass; HOMA, Homeostatic Model Assessment; HbA1c, Glycated Hemoglobin; HDL, High-Density Lipoprotein; LDL, Low-Density Lipoprotein; AST, Aspartate Aminotransferase; ALT, Alanine Aminotransferase; GGT, Gamma-Glutamyl Transferase; CAP, Controlled Attenuation Parameter; FIB-E, Fibroscan Elastography; GFR, Glomerular Filtration Rate.

Figure S1. Stacked ^{1}H CPMG NMR spectra of the analyzed serum samples, showing an expanded view of the aromatic region (approximately 6.7–8.5 ppm).

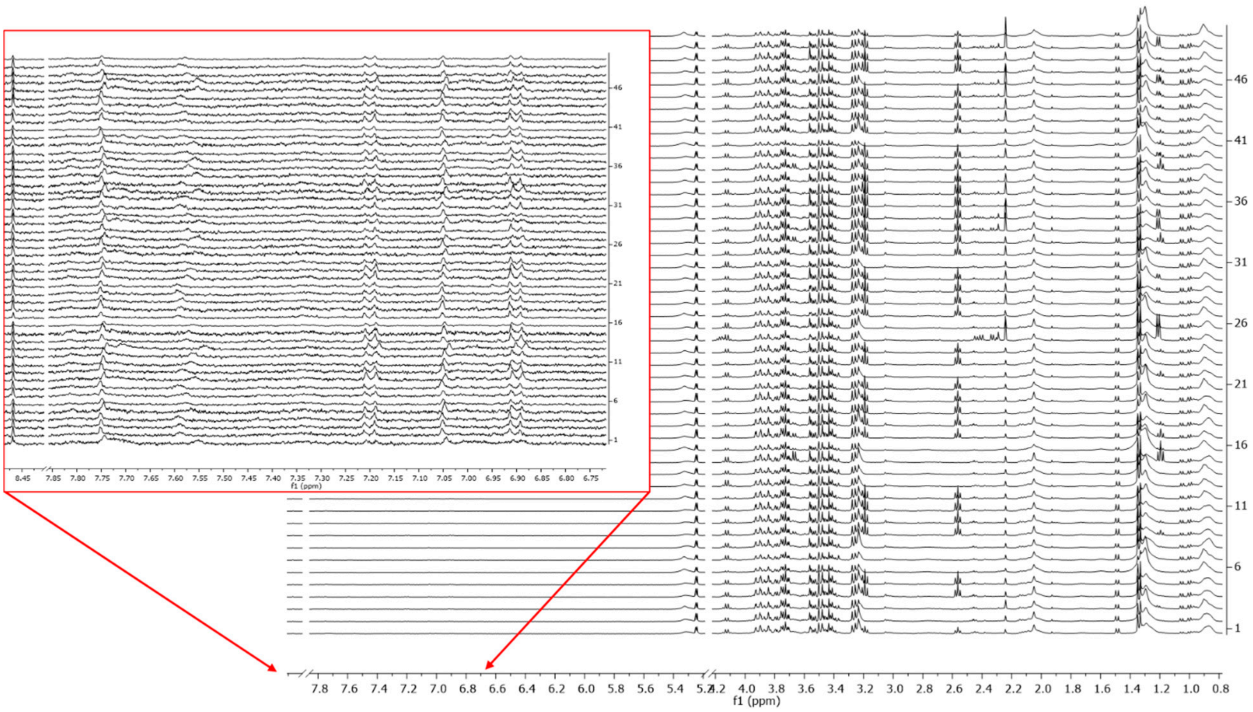

Figure S2. Representative ^{1}H CPMG NMR spectrum of a serum sample acquired at 400 MHz. Peak assignments are based on the chemical shift values reported in Table S1.

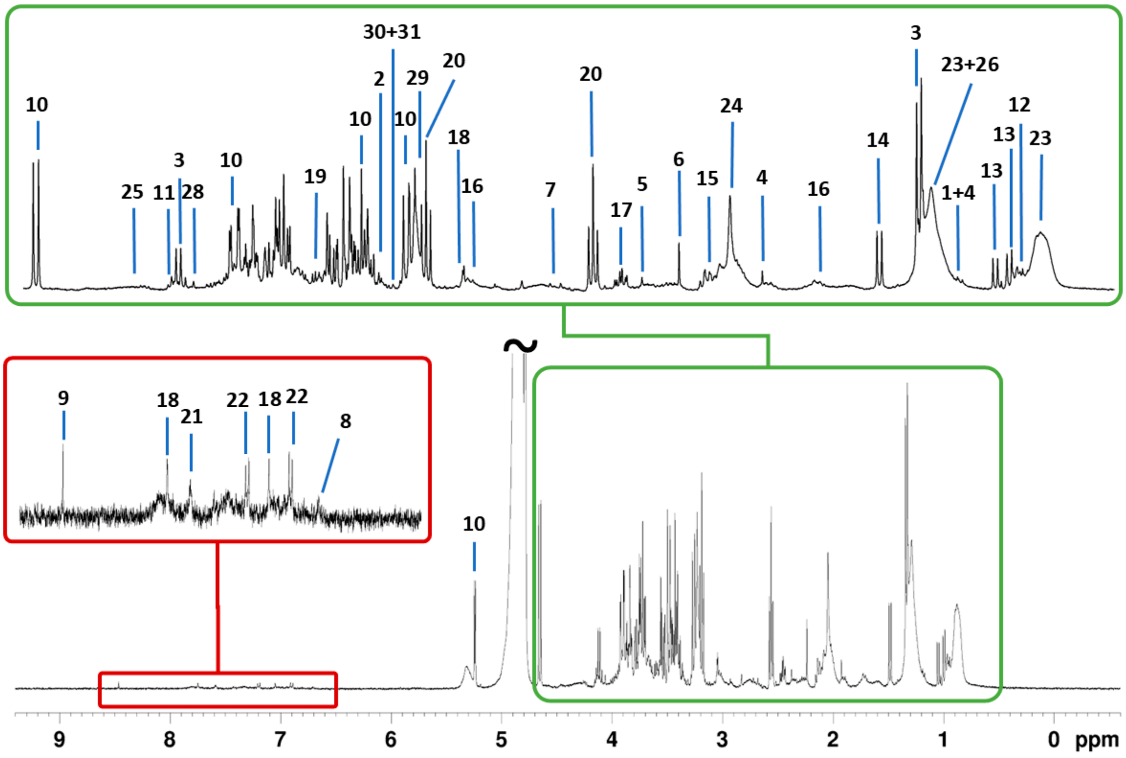

Table S2. List of metabolites contained in the aqueous extracts of serum samples and identified by 1D ^{1}H CPMG experiment.

Compound ID	Compound	δ (ppm)	Multiplicity	*J* (Hz)
Alcohols				
1	**Ethanol**	1.20	t	6.5
		3.67	q	6.5
2	**Methanol**	3.37	s	
Organic acids				
3	**Lactic acid**	1.34	d	6.9
		4.12	q	6.9
4	**β-Hydroxybutyric acid**	1.21	d	6.3
		2.31	dd	6.4; 14.3
		2.42	dd	7.3;14.3
		4.16	m overlapped	
4	**Acetic acid**	1.93	s	
5	**Pyruvic acid**	2.38	s	
6	**Acetoacetate**	2.29	s	
7	**Citric acid**	2.54	d	16.2
		2.70	d	16.1
8	**Fumaric acid**	6.70	s	
9	**Formic acid**	8.47	s	
Carbohydrates				
10	**Glucose**	3.26	dd	9.1; 7.9
		3.43	m	
		3.48	m	
		3.56	m	
		3.75	m	
		3.83	m	
		3.91	dd	12.3; 2.1
		4.66	hidden	
		5.24	d	3.7
11	**Myo-inositol**	3.87	t	2.3
		4.12	s overlapped	
Amino Acids				
12	**Leucine**	0.95	dd overlapped	
		1.72	m	
13	**Valine**	1.00	d	7.0
		1.05	d	7.0
14	**Alanine**	1.49	d	7.3
		3.79	q	7.3
15	**Glutamine**	2.14	td overlapped	
		2.46	td	14.4, 6.8
		3.78	t overlapped	
16	**Lysine**	1.73	m	
		1.90	m	
		3.03	t overlapped	
		3.70	t overlapped	
17	**Glutamic acid**	2.11	m	
		2.41	td	7.4; 2.5
		3.79	td overlapped	
18	**1-Methylhistidine**	3.10	dd overlapped	
		3.20	dd overlapped	
		3.72	s	
		3.94	dd	7.55, 4.92
		7.04	s	
		7.74	s	
19	**Glycine**	3.54	s	

**20**	**β-alanine**	2.56	t	6.7
		3.17	t	6.7
**21**	**Phenylalanine**	7.56	m	
**22**	**Tyrosine**	3.93	t overlapped	
		3.12	dd overlapped	
		2.92	dd overlapped	
		6.90	d	8.5
		7.20	d	8.5
Other				
**23**	**LDL/VLDL**	0.87	br signal	
		1.29	br signal	
**24**	**Unsaturated lipid**	2.05	br signal	
		5.33	br signal	
**25**	**Glycerophosphocholine**	3.24	br signal	
		3.61	m	
		3.74	m	
		4.25	m	
**26**	**N-acetyl glycoproteins (Nac)**	2.05		
**27**	**Acetone**	2.24	s	
**28**	**Creatine/Creatinine**	3.05	s	
		4.06	s	
**29**	**Choline**	3.16	s	
		3.50	dd overlapped	
		4.05	m	
**30**	**Trimethylamino-N-oxide (TMAO)**	3.25	s	
**31**	**Carnitine**	3.23	br s	

[a]singlet (s), doublet (d), triplet (t), quartet (q) doublet of doublets (dd), doublet of triplets (dt), multiplet (m).

Figure S3. Results of the PLS-DA applied to the ^{1}H CPMG NMR spectra by using UV-scaled 0.002 ppm-sized bucketing.

(a) PLS-DA scores plot of ^{1}H CPMG NMR data showing samples at T0 (red circles) and T1 (green triangles). Confidence ellipses (95%) illustrate partial separation between groups. (b) Permutation test (n = 1000) assessing model validity. The observed statistic (red arrow) falls within the distribution of permuted models, yielding a non-significant result ($p = 0.996$), indicating lack of model robustness and absence of statistically valid class discrimination.

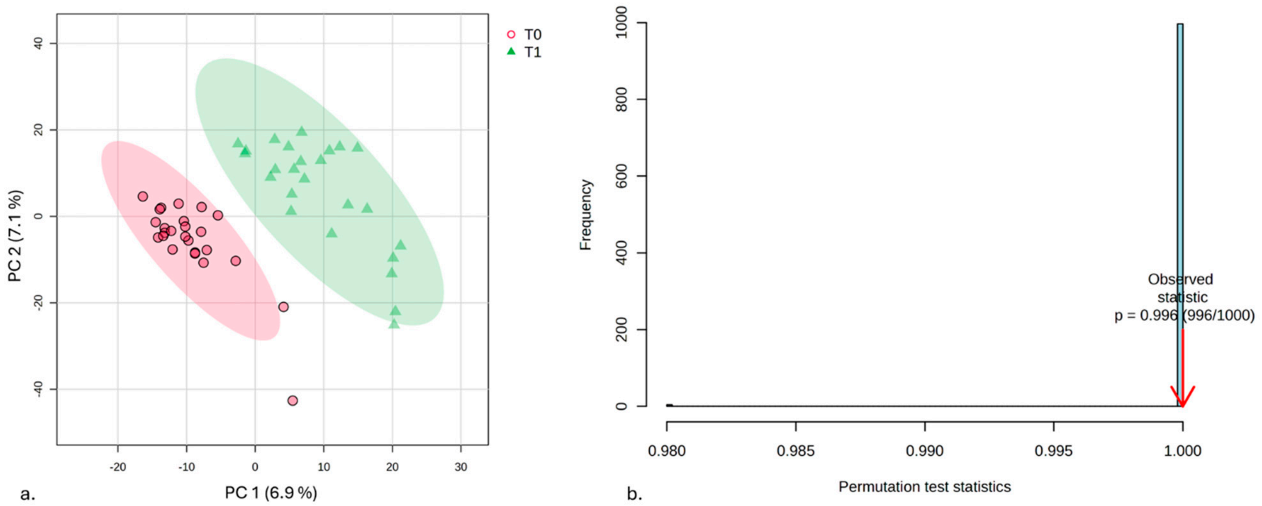

Table S3. Analytical performance parameters for targeted metabolite quantification derived from ^{1}H CPMG NMR spectra.

Signal-to-noise ratio (S/N), limits of detection (LOD), and limits of quantification (LOQ) were determined from calibration curves using standard solutions. LOD and LOQ were calculated according to standard analytical criteria ($LOD = 3\times\sigma/S$, $LOQ = 10\times\sigma/S$), where σ represents the standard deviation the of the residuals of the calibration curve and S the slope of the calibration curve. Signal-to-noise ratios (S/N) are reported as descriptive indicators of signal quality across the calibration range and are not used for the calculation of LOD and LOQ, which were determined from calibration curve statistics. Calibration curves showed excellent linearity for both metabolites ($R^2 > 0.999$), supporting the robustness of the quantitative analysis.

Metabolite	Chemical Shift (ppm)	Selected Signal	R^2	S/N (Range)	LOD (ppm)
Tyrosine	6.88–6.74	Aromatic protons	0.9993	6.6–112.7	9.11
β-Alanine	2.44–2.50	CH_2 protons	0.9990	30.8–598.8	24.41
